# Comparative transcriptome sequencing of germline and somatic tissues of the *Ascaris suum *gonad

**DOI:** 10.1186/1471-2164-12-481

**Published:** 2011-10-01

**Authors:** Xuan Ma, Yingjie Zhu, Chunfang Li, Yunlong Shang, Fanjing Meng, Shilin Chen, Long Miao

**Affiliations:** 1 Noncoding RNA laboratory, National Laboratory of Biomacromolecules, Institute of Biophysics, Chinese Academy of Sciences, Beijing 100101, China; 2 Institute of Medicinal Plant Development, Chinese Academy of Medical Sciences, Beijing 100094, China; 3 Linyi Chest Hospital, Linyi 276034, Shandong Province, China

## Abstract

**Background:**

*Ascaris suum *(large roundworm of pigs) is a parasitic nematode that causes substantial losses to the meat industry. This nematode is suitable for biochemical studies because, unlike *C. elegans*, homogeneous tissue samples can be obtained by dissection. It has large sperm, produced in great numbers that permit biochemical studies of sperm motility. Widespread study of *A. suum *would be facilitated by more comprehensive genome resources and, to this end, we have produced a gonad transcriptome of *A. suum*.

**Results:**

Two 454 pyrosequencing runs generated 572,982 and 588,651 reads for germline (TES) and somatic (VAS) tissues of the *A. suum *gonad, respectively. 86% of the high-quality (HQ) reads were assembled into 9,955 contigs and 69,791 HQ reads remained as singletons. 2.4 million bp of unique sequences were obtained with a coverage that reached 16.1-fold. 4,877 contigs and 14,339 singletons were annotated according to the *C. elegans *protein and the Kyoto Encyclopedia of Genes and Genomes (KEGG) protein databases. Comparison of TES and VAS transcriptomes demonstrated that genes participating in DNA replication, RNA transcription and ubiquitin-proteasome pathways are expressed at significantly higher levels in TES tissues than in VAS tissues. Comparison of the *A. suum *TES transcriptome with the *C. elegans *microarray dataset identified 165 *A. suum *germline-enriched genes (83% are spermatogenesis-enriched). Many of these genes encode serine/threonine kinases and phosphatases (KPs) as well as tyrosine KPs. Immunoblot analysis further suggested a critical role of phosphorylation in both testis development and spermatogenesis. A total of 2,681 *A. suum *genes were identified to have associated RNAi phenotypes in *C. elegans*, the majority of which display embryonic lethality, slow growth, larval arrest or sterility.

**Conclusions:**

Using deep sequencing technology, this study has produced a gonad transcriptome of *A. suum*. By comparison with *C. elegans *datasets, we identified sets of genes associated with spermatogenesis and gonad development in *A. suum*. The newly identified genes encoding KPs may help determine signaling pathways that operate during spermatogenesis. A large portion of *A. suum *gonadal genes have related RNAi phenotypes in *C. elegans *and, thus, might be RNAi targets for parasite control.

## Background

The genus *Ascaris*, also known as the "giant intestinal roundworms", contains the largest intestinal nematode species. *Ascaris lumbricoides *causes the commonest helminth infection of humans, whereas a closely related species, *Ascaris suum*, typically infects pigs and causes substantial financial losses to the meat industry. The *A. suum *female is capable of producing more than 200,000 eggs per day and these eggs can survive and remain infective after many years in soil [1]. At present, there is no effective alternative to chemical control of intestinal parasites and resistance to anthelmintics has become an emerging problem [2]. Greater knowledge of nematode biology is urgently needed to enable the development of new biotechnological tools (*e.g*., RNA interference) for parasite control.

To better understand the molecular and biochemical basis of nematode development, nematode EST projects have generated more than 250,000 ESTs from 30 species, including *A. suum *[3]. Large-scale EST datasets have also been acquired by next-generation sequencing (NGS) technologies and the associated bioinformatic pipeline has been developed [4,5]. This vast collection of ESTs combined with the extensive knowledge of *Caenorhabditis elegans *biology provides opportunities to elucidate functionally conserved mechanisms in nematode biology. Employment of NGS technologies has greatly accelerated the 959 Nematode Genomes project http://www.nematodes.org/nematodegenomes/index.php/Main_Page. Genome sequencing of *A. suum *somatic cells is ongoing http://www.sanger.ac.uk/resources/downloads/helminths/ascaris-suum.html, and a draft genome and transcriptome of *A. suum *is now available http://www.nematode.net/NN3_frontpage.cgi?navbar_selection=home&subnav_selection=asuum_ftp. To date, there have been 38 *A. suum *EST libraries with ~55,000 sequences available in the NEMBASE4 database http://www.nematodes.org/nembase4/ and these ESTs were obtained using conventional cDNA library sequencing technology.

In the present study, we applied 454 pyrosequencing technology to unravel the transcriptome of the male *A. suum *gonad, the organ for reproduction. *A. suum *males have a large gonad that can be readily isolated by dissection to provide large numbers of sperm that are suitable for biochemical and cell biological studies [6]. The male *A. suum *gonad is composed of three distinct regions; the testis and seminal vesicle form germline tissue and the glandular vas deferens forms somatic tissue. Sperm are stored in the seminal vesicle. During copulation, the spherical, non-motile sperm are activated into bipolar, amoeboid spermatozoa by an unknown component secreted by the glandular vas deferens. The motility of amoeboid sperm is driven by the regulated assembly and disassembly of major sperm protein (MSP) cytoskeleton [7,8]. The mechanism of sperm activation is poorly understood and the details of MSP-based sperm motility are yet to be determined, although several proteins (*e.g*., MPOP, MFPs and PP2A) that participate in the dynamics of the MSP cytoskeleton have been identified [9-12]. Despite the advantages of large gametes and the easy isolation of reproductive fluids from *A. suum*, there have been few studies focusing on sperm chromatin or on distinctions between germline and somatic tissues in *A. suum*. In addition, chromatin diminution in *A. suum *represents a fascinating exception to the general rule of the constancy of the genome. However, the complex mechanism of this phenomenon, involving DNA degradation and new telomere addition remain an enigma [13-18]. One of the barriers to answering the above questions is the lack of gene expression data for the reproductive tissues of *A. suum*.

To facilitate diverse studies concerning reproductive biology in *A. suum*, we acquired the transcriptomes of germline and somatic tissues of *A. suum *gonad using the RNA-seq approach. Comparison of these two tissues showed that the nucleic acid metabolic and proteasome-ubiquitin pathways are more active in the germline than in the soma. Further comparison with *C. elegans *microarray data identified 165 conserved germline-enriched genes in *A. suum*. We also categorized the RNAi phenotypes for *A. suum *gonadal genes, taking advantage of the *C. elegans *RNAi phenotype database. Therefore, these *A. suum *transcriptome data provide a valuable platform for both fundamental biological studies (*e.g*., MSP-based sperm motility and spermatogenesis studies) and for research concerning parasite control (*e.g*. use of RNAi).

## Results

### 454 sequencing and *de novo *assembly of *A. suum *gonad transcriptome

**Figure 1:**
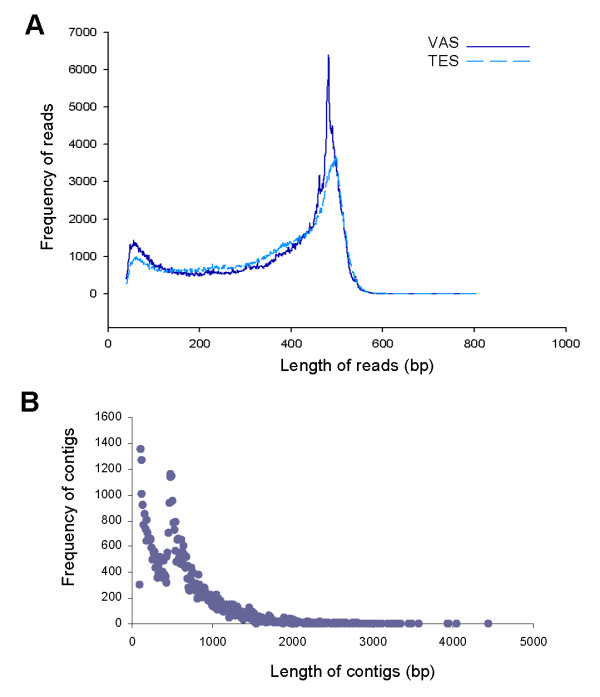
**Size distribution of 454 raw sequencing reads and assembled contigs**. **(A) **Raw reads. **(B) **Assembled contigs. TES and VAS represent germline and somatic tissues of the *A. suum *gonad, respectively.

The male *A. suum *gonad was dissected into two parts: testis and seminal vesicle (TES) and glandular vas deferens (VAS), and both samples were subjected to total RNA extraction followed by cDNA synthesis. Second-strand cDNAs with trimmed poly(A) tails were used for high-throughput sequencing on a 454 GS FLX Titanium platform. We performed two runs that produced ~1.16 million raw reads constituting a total of ~0.4 billion base-pairs (bp). The majority of the reads were over 400 bp and the average length of the reads was ~356 bp. The size distribution of the raw reads from both samples is shown in Figure 1A. To acquire high-quality reads, we filtered out the reads shorter than 50 bp, which account for 4.3% of total reads. These high-quality reads were then used in *de novo *assembly using Newbler (Version 2.3). 999,214 reads from either TES or VAS were assembled into 9,955 contigs. These contigs range from 100 bp to 6,649 bp and 97.6% of them were assembled from three or more reads. The size distribution of the contigs is shown in Figure 1B. The number of singletons in TES and VAS datasets was 30,137 and 39,654, respectively, and they together comprised 10% of total reads. We obtained 2.4 Mbp of unique sequences with a coverage that reached 16.1-fold. All unique sequences are available at http://159.226.118.206/miaolab/index.htm. The contigs longer than 200 bp have been deposited in the GenBank Transcriptome Shotgun Assembly (TSA) database under the accession numbers JO467643-JO475858. A summary of 454 sequencing and assembly is shown in Table 1.

**Table 1:** Summary of 454 sequencing and assembly

	TES*	VAS*
**Sequencing**		
No. of reads	572,982	588,651
No. of base-pairs	204,129,803	209,849,741
Average read length (bp)	356.3	356.5

**Assembly**		
No. of singletons (> 50 bp)	30,137	39,654
Average length of singletons	287.6	251.5
No. of contigs	9,955	
Average length of contigs (bp)	621.8	

Total coverage (bp)	24,828,337	

*Note: TES and VAS represent germline and somatic tissues of *A. suum *gonad, respectively.

The current *A. suum *testis EST library in NEMBASE4 has collected 2,868 ESTs. These ESTs correspond to 595 homologous genes in *C. elegans *(BLAST cutoff E-value = 1e^-5^). In contrast, our *A. suum *gonad transcriptome corresponds to 4,207 homologous genes in *C. elegans *and 3,686 novel gonadal genes were identified (Additional file 1). This suggests our *A. suum *gonad transcriptome has a deeper coverage than the conventional EST library.

### Functional assignments of *A. suum *454 sequencing data

To annotate the *A. suum *454 transcriptome data, we compared all unique sequences against the *C. elegans *protein database in WormBase, as well as against the Kyoto Encyclopedia of Genes and Genomes (KEGG) protein databases using BLASTX (cutoff E-value = 1e^-5^). A total of 4,877 contigs (49%) and 14,339 singletons (20.5%) were annotated. A large portion of the 454 sequences have not been functionally defined. Some sequences can be annotated by increasing the E-value and others may represent *A. suum *specific genes. In summary, 9,822 unique sequences (corresponding to 5,683 gene models) were annotated in the TES dataset and 12,123 unique sequences (corresponding to 4,122 gene models) were annotated in the VAS dataset (Additional file 2). Although ~2,000 more sequences were assigned in the VAS dataset compared with the TES dataset, TES has ~1,500 more gene models than VAS suggesting that there are more diverse genes expressed in TES than in VAS tissues.

Tubulin genes (> 12,000 reads) are the most abundant transcripts in the *A. suum *gonad; the fibulin genes (> 10,000 reads), whose activity is essential for gonad and body morphology in *C. elegans *[19], are also highly abundant. The expression of genes encoding intermediate filament proteins, heat shock proteins, ribosomal proteins, aldehyde reductase and major sperm proteins were also enriched. It should be noted that among the 100 most highly enriched genes, over half have not been functionally characterized.

### Metabolic pathway mapping

**Figure 2:**
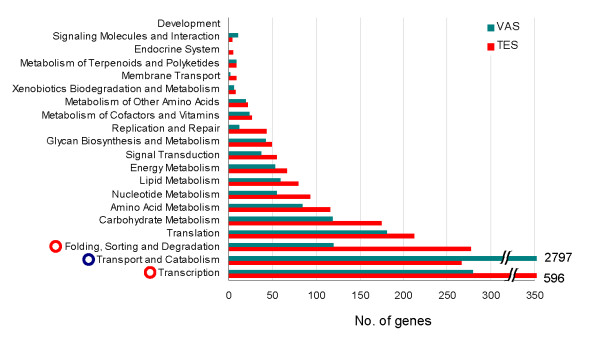
**Distribution of the number of genes expressed in various metabolic pathways**. Red and green bars represent the TES and VAS datasets, respectively. Different metabolic pathways are shown on the left. Blue circle marks the metabolic process requiring the most number of genes in VAS tissues; red circles mark the processes that require twice as many genes in TES compared with VAS tissues.

To gain insight into *A. suum *gonad metabolic pathways, we mapped the *C. elegans *homologues of *A. suum *genes to the KEGG pathways. A total of 5,426 unique *A. suum *sequences corresponding to 850 homologous genes in *C. elegans *were assigned to metabolic pathways (Additional file 3). Among the 5,426 sequences, 33.1% were expressed in TES and 77.9% were expressed in VAS; only 11.1% were expressed in both TES and VAS. This suggested TES and VAS express distinct groups of genes that participate in their respective metabolic processes. As shown in Figure 2, the majority of TES and VAS genes are classified into pathways for transcription, transport and catabolism, folding, sorting and degradation, translation, carbohydrate and amino acid metabolism. In VAS, a large number of genes participate in the transport and catabolism pathway (highlighted by blue circle), while TES has twice the number of genes involved in the transcription pathway and the folding, sorting and degradation pathway as VAS (highlighted by red circles).

### Comparative analysis of TES and VAS datasets

**Figure 3:**
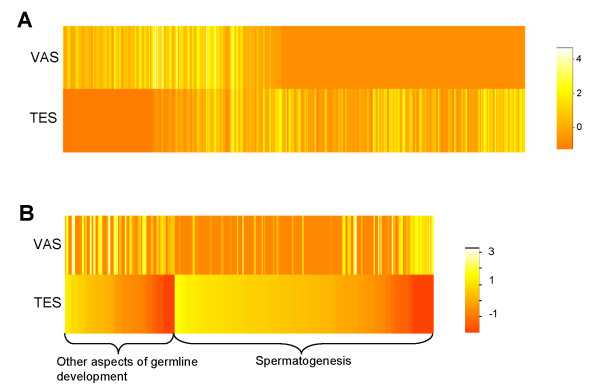
**Digital gene expression profiling of all contigs and the germline-enriched contigs**. **(A) **Heat-map showing the global gene expression of all contigs. **(B) **Heat-map showing the expression of the germline-enriched contigs. Rows represent TES and VAS, each column represents a different contig. Relative gene abundance is defined by log_10 _of the normalized read number followed by Z-score transformation to visualize the expression level. Yellow indicates higher expression; red indicates lower expression.

Annotation of the TES and VAS datasets has indicated differences in gene expression in a tissue-specific manner. The majority of TES tissues comprise germ cells while VAS tissues are exclusively composed of somatic cells. To further characterize their differential gene-expression patterns, we used the contig dataset to compare their transcriptomes according to the number of reads, and a global expression profiling for all contigs is shown in Figure 3A. These two tissues have dramatically different expression patterns. To quantify these differences, we normalized the expression levels of TES and VAS before calculating the reads-ratio of TES to VAS. The thresholds 10 and 0.1 were set to identify the highly expressed contigs in TES and VAS, respectively. This analysis identified 3,110 contigs having levels of expression that were at least 10-fold higher in TES compared with VAS, and 1,165 contigs whose levels of expression were at least 10-fold higher in VAS than in TES (Additional file 4).

To highlight the functions of differentially expressed genes between TES and VAS, the contigs having 10-fold higher levels of expression were searched against the STRING database http://string-db.org/ to identify the functional associations of these genes. The result demonstrated that, compared with the somatic VAS tissues, the germline TES tissues has a more complex gene/protein interaction network (Additional file 5). In the germline, the genes encoding proteins involved in DNA replication and RNA transcription are highly enriched; the germline also expresses a large number of genes participating in the proteasome and ubiquitin-mediated proteolysis pathways. These data underpin the nature of the germline, which functions through cell cycle progression and differentiation. It also shows the necessity of the proteasome in germline development. In addition, as expected, the expression of genes encoding MSPs and sperm specific proteins was highly enriched in the germline.

To validate the gene expression changes observed between TES and VAS tissues, we selected 18 genes (*Daf-21*, *Cul-1*, *Skr-1*, *Ubc-7*, *Rbx-1*, *Rpn-1*, *Pas-4*, *Pbs-2*, *Let-70*, *Eel-1*, *Kin-19*, *Sel-12*, *Paa-1 Gsk-3*, *Cdc-42*, *Smo-1*, *Exos-7 *and *Pri-1*) having significantly higher levels of expression in TES than in VAS for semi-quantitative RT-PCR analysis. These genes are involved in processes including, protein processing, ubiquitin-proteasome pathways, Wnt signaling, cell division and nucleic acid metabolism. The results (Additional file 6) showed that the expression in the majority of these genes is either down-regulated or absent in VAS as compared with TES.

### Comparison with *C. elegans *microarray and RNAi screening datasets

Germline development in *C. elegans *has been extensively studied http://www.wormbook.org/toc_germline.html. *A. suum *and *C. elegans *belongs to Clade III and V, respectively, in the phylum Nematoda, and it is estimated that these two clades have an evolutionary divergence of 350 million years [20]. To identify the conserved genes regulating gonad development, we compared the *A. suum *gonad transcriptome with two *C. elegans *datasets acquired by microarray and genome-wide RNAi analyses.

Using microarray technology, Reinke *et al*. identified 1,092 and 340 genes that have enriched expression in *C. elegans *adult male germline and soma, respectively (14 of these genes are pseudogenes or are no longer available in WormBase) [21]. BLAST analysis showed that 532 (49.1%) of the *C. elegans *germline-enriched genes and 139 (32.8%) of the soma-enriched genes have homologues in *A. suum *TES and VAS tissues, respectively (Additional file 7). The corresponding *A. suum *germline-enriched genes include 259 contigs and 37 singletons and the expression profiling of the 259 contigs is illustrated in Figure 3B. 165 genes of these contigs have over 10-fold higher levels of expression in TES than in VAS, and thus might represent conserved genes controlling germline development in different nematode species (Additional file 8). Among them, 137 genes (83%) are spermatogenesis-enriched and the rest are involved in other aspects of germline development (*e.g*., mitotic proliferation). Substantial numbers of serine/threonine kinases and phosphatases (KPs), as well as tyrosine KPs were identified, suggesting pivotal roles of phosphorylation during spermatogenesis. It should be noted that the genes encoding KPs are over-represented among the sperm-enriched genes in *C. elegans *[22].

**Figure 4:**
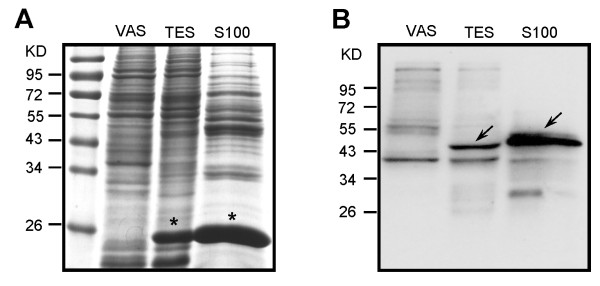
**Western blot analysis of tyrosine phosphorylation (pY) in protein extracts of VAS, TES and sperm**. **(A) **SDS-PAGE of total protein extracts of VAS, TES and sperm. Asterisks point to the MSPs that exist exclusively in sperm. **(B) **Western blot analysis of VAS, TES and sperm protein extracts probed with anti-pY. Arrows indicate a ~45 KD protein that is abundant in sperm extracts (S100).

When comparing the TES and VAS datasets, we noticed an enriched expression of genes encoding KPs in TES tissues. There are 242 contigs in the TES dataset encoding KPs that have levels of expression 10-fold higher than those in VAS; in contrast, there are only 17 contigs encoding KPs with VAS/TES expression ratios above 10 (Additional file 9). The abundance of genes encoding KPs in TES tissues prompted us to examine the level of phosphorylation in total protein extracts from TES and VAS. Western blot analysis (Figure 4) showed that a ~45 KD protein (marked by arrowhead) in testis and sperm is associated with strong tyrosine phosphorylation (pY). The comparable amount of MSPs (marked by asterisk) in TES and sperm extracts (S100) indicates that this ~45 KD protein is abundant in sperm. In all soluble proteins of the sperm, this protein has the highest pY level suggesting that it may have essential functions during spermatogenesis or spermiogenesis. In contrast, the phosphorylated form of this protein was not detected in the vas deferens.

**Figure 5:**
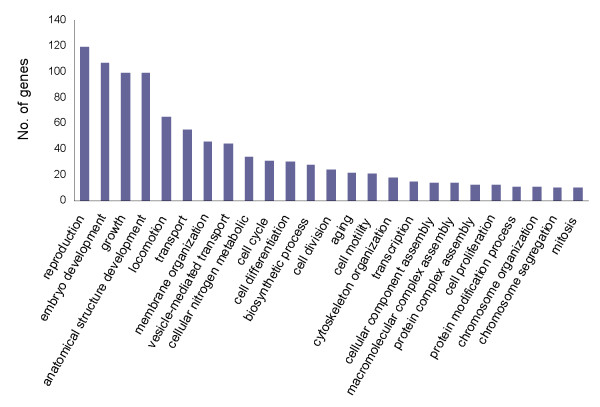
**Gene Ontology (GO) categorization of gonadal genes**. According to the *C. elegans *dataset, 140 *A. suum *genes were identified to control gonadal development, and GO classification of these genes is illustrated. Categories with fewer than 10 genes are not shown.

Through a genome-wide RNAi screen, Kalis *et al*. identified 207 genes which are essential for gonadogenesis in *C. elegans *(3 genes are no longer available in WormBase) [23]. BLAST analysis showed that 148 (71.5%) of these genes have homologues represented in the *A. suum *gonad transcriptome (Additional file 10). This corresponded to 140 *A. suum *genes and gene ontology (GO) analysis showed most of them regulate reproduction, embryo development, growth, locomotion and development of anatomical structures, as shown in Figure 5.

### Categorization of RNAi phenotypes of *A. suum *gonadal genes

**Figure 6:**
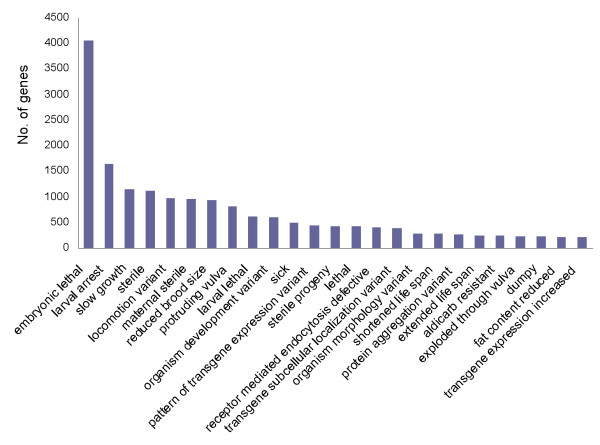
**RNAi phenotypic categorization of *A. suum *annotated gonadal genes**. 2,681 *A. suum *gonadal genes were identified to have associated RNAi phenotypes according to the *C. elegans *RNAi phenotype database. Categories with fewer than 200 genes are not shown.

RNA interference (RNAi) was first discovered in *C. elegans *[24] and has since been widely used to suppress gene expression in a variety of organisms. Effective RNAi on *A. suum *larval development has been reported [25]. Therefore, we used the 11,968 genes that are orthologous between *C. elegans *and *A. suum *to query the *C. elegans *RNAi phenotype database. RNAi phenotypes associated with 2,681 genes were identified (Additional file 11). The main RNAi phenotypes are presented in Figure 6. These data show that a large portion of these genes are associated with embryonic lethality, slow growth, larval arrest, sterility and locomotion defects. This is in line with the functional classifications of the *A. suum *gonadal genes shown in Figure 5.

## Discussion and Conclusions

Nematodes are one of the most diverse phyla and they make up approximately 80% of all individual animals on earth [3,26]. As the most prevalent nematode parasite in pigs, *A. suum *causes massive losses to the swine industry worldwide. The 959 Nematode Genomes Project has included this species for whole-genome sequencing and a draft genome and transcriptome of *A. suum *has just become available. In this study, we adopted 454 sequencing technology to determine the transcriptome of the *A. suum *gonad so as to facilitate further studies of this organism.

A total of 0.4 billion bp were obtained by 454 sequencing, which were assembled into 25 million bp, which is equivalent to the *C. elegans *exome (26 million bp). 86% of the high-quality reads were assembled into longer contigs, suggesting that these sequencing data had a high coverage. We annotated half of the contigs and 20% of the singletons; a large fraction of sequences have not been functionally assigned. The unannotated 454 ESTs may contain precursor non-coding RNAs (*e.g*., pre-miRNA, pre-snoRNAs) as well as the polyadenylated ncRNA classes; for example, over 13% and 26% of full-length cDNAs in mice and human, respectively, are proposed to be polyadenylated mRNA-like ncRNAs [27-29]. The germline (TES) encompasses more gene models than the soma (VAS). Metabolic pathway mapping analysis also showed that TES and VAS datasets have distinct groups of genes involved in their respective metabolic processes.

To investigate germline-soma distinctions, we compared the digital transcriptomes of TES and VAS, and identified numerous TES-specific pathways, including DNA replication and proteasome and ubiquitin-mediated proteolysis pathways. These pathways might be required to regulate germline proliferation and differentiation. The proteasome has been documented to regulate the balance between cell proliferation and meiotic entry in *C. elegans *[30]. The genes encoding MSPs, sperm specific proteins and pyruvate dehydrogenase were the most highly expressed genes in the *A. suum *germline. MSP comprises 10-15% of the total proteins in nematode sperm [31] and sperm motility is driven by the regulated assembly and disassembly of MSP [7,8,32,33]. Hence, the high levels of expression of MSP genes were expected. With regard to pyruvate dehydrogenase, we speculate it might promote the tricarboxylic acid (TCA) cycle to supply energy for germ cell development.

We have a particular interest in *A. suum *spermatogenesis. Based on the *C. elegans *microarray data, 165 *A. suum *genes (83% are spermatogenesis-enriched) were identified as conserved genes controlling germline development. The most abundant proteins involved in *A. suum *spermatogenesis consist of sperm specific proteins, PDZ domain proteins, tyrosine kinases and phosphatases (KPs), and serine/threonine KPs. Identification of the genes encoding KPs in this analysis underpins the essential role of phosphorylation in the regulation of spermatogenesis. As the most common posttranslational modification, phosphorylation has been established to link to sperm function in a variety of species. In mammalians, the processes regulated by phosphorylation include capacitation, hyperactivated motility, zona pellucida binding, acrosome reaction and sperm-oocyte binding and fusion [34-36]. In *C. elegans*, the genes encoding KPs are over-represented among the sperm-enriched genes [22]. Clues to the phosphorylation signaling pathway that controls MSP-based cell motility were also documented in *A. suum*. A phosphorylated membrane protein (named MPOP) recruits a soluble casein kinase 1 (named MPAK) to the inner leaflet of the plasma membrane to initiate sperm motility [37]; MPAK, in turn, phosphorylates a second cytosolic protein (named MFP2) to accelerate MSP assembly [10,11]. A putative PP2A homologue was shown to trigger the retraction of MSP cytoskeleton [12]. The newly identified KPs in this study may aid in determining the signaling pathways that operate during spermatogenesis in *A. suum*. We provide evidence that a ~45 KD protein in sperm is associated with strong tyrosine phosphorylation (pY). Because tyrosine phosphorylation and dephosphorylation act as a molecular switch to regulate MSP assembly [33], we propose this ~45 KD protein may be involved in MSP-based sperm motility. Immunolabeling of pY on the leading edge of spermatozoa has also been observed (Zhao Y. and Miao L., unpublished observations), which reinforces the notion that phosphorylation plays a role in *A. suum *spermatogenesis/spermiogenesis.

Lastly, *C. elegans *genes that are homologous to genes represented in the *A. suum *gonad transcriptome were examined for associated RNAi phenotypes. Although the genes involved in spermatogenesis are possibly insensitive to RNAi [38], a variety of RNAi phenotypes, mostly embryonic lethality, arrested growth or sterility, were retrieved from the *C. elegans *database. This RNAi phenotypic categorization is in line with the functional classification of the gonad developmental genes, the majority of which control reproduction, embryo development and growth. Due to the growing concern of anthelminth resistance, RNAi provides a new means to combat parasitic nematodes. RNAi has been successfully used to knock down target genes in a few parasitic nematode species, including *B. malayi *[39], *H. glycines *[40], *G. pallida *[40], *O. volvulus *[41], *T. colubriformis *[42] and notably, *A. suum *[25]. Recently, serine/threonine phosphatases have been recommended as targets for new nematicidal drugs [43]. Therefore, we anticipate that these *A. suum *gonad transcriptome sequencing data will provide opportunities to use RNAi as a novel anti-parasite agent for parasite control.

## Methods

### Collection of *A. suum *gonad samples

*A. suum *males were collected from the intestines of infected hogs at Zhongrui Pork Processors (Liucun, Beijing, China) and were stored in worm buffer (phosphate buffered saline containing 100 mM NaHCO_3_, pH 7.0, 37°C). *A. suum *gonads were dissected into two parts: (1) testis and seminal vesicle; (2) glandular vas deferens. Dissected samples were immediately frozen in liquid nitrogen prior to storage at -80°C.

### cDNA synthesis and 454 pyrosequencing

Total RNAs from *A. suum *testis and vas deferens were prepared using TRIzol reagent (Invitrogen, Carlsbad, CA, USA) before removal of trace genomic DNA using DNAse I (Promega, Madison, WI, USA). Poly A^+ ^RNA was purified using an Oligotex Direct mRNA Kit (Qiagen, Hilden, Germany) followed by first and second-strand cDNA synthesis using the Universal RiboClone cDNA Synthesis System (Promega) with a modification as follows. We designed a poly(T) adaptor with a *Bsg*I site flanking the poly(T) sequence (5'-CGTGTGCAGT_(20)_VN-3') for cDNA synthesis. The purified second-strand cDNAs were digested by *Bsg*I and recovered with a QIAquick PCR Purification Kit (Qiagen). Both double-strand cDNA samples were subjected to 454 pyrosequencing using the GS FLX Titanium Kit.

### 454 sequencing data analyses

High-quality sequences (> 99.5% accuracy on single base reads) were filtered to remove short ESTs (< 50 bp) before assembly using Newbler (version 2.3). For assembly, the quality score threshold was set to 40. All unique sequences containing both contigs and singletons were compared against the *C. elegans *confirmed protein database (derived from Wormbase) as well as the KEGG protein databases for all organisms http://www.genome.jp/kegg/download/ by BLASTX; the BLAST cutoff E-value was set at < 1e^-5^. The *A. suum *gonad developmental genes were functionally assigned by GO Slimer http://amigo.geneontology.org/cgi-bin/amigo/slimmer?session_id. The *A. suum *genes were mapped onto metabolic pathways according to *C. elegans *pathways http://www.genome.jp/kegg/download/. Total read numbers of TES and VAS datasets were normalized to equal levels, and the relative gene abundance was defined by log_10 _of the normalized read number. Heat-maps were generated using R software (version 2.12.0).

### Reverse Transcription PCR

Total RNAs from *A. suum *testis and vas deferens were isolated before being reverse transcribed into cDNA using the SuperScript III First-Strand Synthesis System (Invitrogen). The cDNA products were diluted 10-fold for use as RT-PCR templates. PCR was performed using High Fidelity PCR SuperMix (TransGen, Beijing, China) under the following conditions: 94°C for 3 min; 30 cycles of 94°C for 30 s, 57°C for 30 s, 72°C for 1 min; 72°C for 8 min. The genes *Act-1 *and *eIF-4A *were used as controls.

### Preparation of TES and VAS protein extracts and of sperm extracts (S100)

Fresh *A. suum *TES and VAS tissues were disrupted in HKB buffer (50 mM Hepes, 65 mM KCl, 10 mM NaHCO_3_, pH 7.1) in a homogenizer. An equal volume of lysis buffer (100 mM Hepes, 300 mM NaCl, 2% Triton-100) was added and samples were then placed on ice for 30 min before centrifugation for 30 min at 20,000 rpm. Supernatants were then collected for Western blot analysis. To prepare sperm extracts (S100), frozen sperm were thawed on ice for 1 hr and centrifuged for 10 min at 13,000 rpm. Supernatant was centrifuged at 100,000 × g for 1 hr at 4°C. Supernatant (S100) was further analyzed by Western blotting.

### SDS-PAGE and Western blotting

SDS-PAGE and Western blotting were performed as previously described [12]. Western blots were probed with anti-phosphotyrosine primary antibody (Millipore, Billerica, MA, USA) at 0.2 μg/mL, followed by peroxidase-conjugated secondary antibody, and developed with enhanced chemiluminescence (PerkinElmer, Waltham, MA, USA).

## Authors' contributions

XM and LM conceived and designed the experiments. SC helped to conceive and coordinate the experiments. FM participated in *A. suum *sample collection. XM prepared ds cDNA samples and performed RT-PCR. CL conducted library construction and 454 pyrosequencing. XM and YS extracted total proteins from TES and VAS tissues and performed Western blot analyses. XM and YZ performed data analyses. XM and LM wrote the manuscript. All authors have read and approved the final manuscript.

## Supplementary Material

Additional file 1 **Summary of gene assignments for the *A. suum *testis EST library in NEMBASE4 and for the *A. suum *gonad transcriptome**. Sheet GenBank EST summarizes the *A. suum *testis EST library; Sheet Gonad 454 data summarizes the *A. suum *gonad transcriptome. Click here for file

Additional file 2 **Annotation of *A. suum *germline (TES) and soma (VAS) transcriptomes**. Click here for file

Additional file 3 **Metabolic pathway assignment of *A. suum *TES and VAS transcriptomes**. Click here for file

Additional file 4 **Transcripts with relatively high expression levels in TES and VAS**. Sheet TES summarizes transcripts having a 10-fold or higher level of expression in TES compared with VAS; Sheet VAS summarizes transcripts having a 10-fold or higher level of expression in VAS compared with TES. Click here for file

Additional file 5 **Gene/Protein association network for genes highly expressed in TES**. The functions of the highlighted gene clusters are as follows: gray, proteasome pathway; yellow, ubiquitin pathway; green, nucleotide metabolism; pink, ribosome members; blue, MSP family. Click here for file

Additional file 6 **RT-PCR verification of highly expressed genes in TES**. Genes *Act-1 *and *eIF-4A *are used as controls. Gene codes are from *C. elegans*. Click here for file

Additional file 7 **Predicted germline and soma-enriched genes in *A. suum***. Datasets were acquired by comparison of the *A. suum *gonad transcriptome with published *C. elegans *microarray data. Click here for file

Additional file 8 **Summary of the 165 *A. suum *candidate genes conserved in nematode germline development**. Click here for file

Additional file 9 **Contigs encoding kinases and phosphatases that are highly expressed in TES and VAS tissues**. Sheet TES summarizes the transcripts of KPs having a 10-fold or higher level of expression in TES compared with VAS; Sheet VAS summarizes the transcripts of KPs having a 10-fold or higher level of expression in VAS compared with TES. Click here for file

Additional file 10 **Genes predicted to control gonadogenesis in *A. suum***. Dataset was acquired by comparison of the *A. suum *gonad transcriptome with published *C. elegans *genes that regulate gonad development. Click here for file

Additional file 11 **RNAi phenotypic categorization of *A. suum *gonadal genes**. Each *A. suum *sequence ID is in bracket. RNAi phenotype codes are from Wormbase. Click here for file
