# Supplementary figures and images for: Comparative transcriptome sequencing of germline and somatic tissues of the Ascaris suum gonad
Source: BMC Genomics. 2011 Oct 1;12:481. doi: 10.1186/1471-2164-12-481 (PMC3203103; doi:10.1186/1471-2164-12-481)

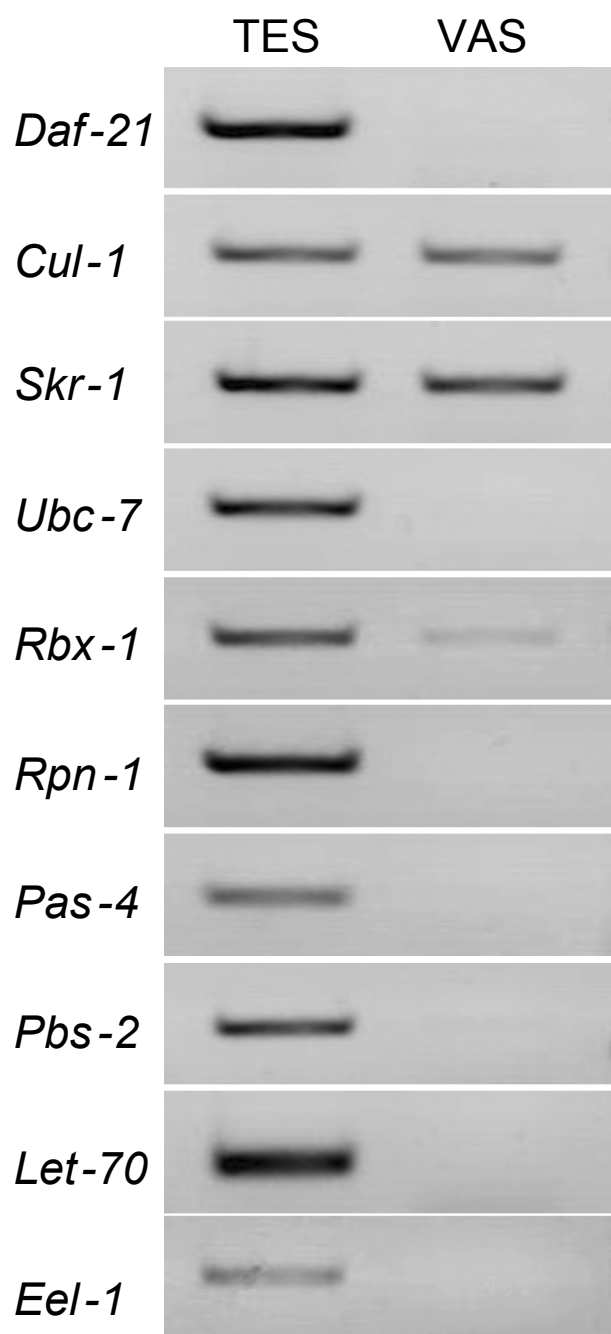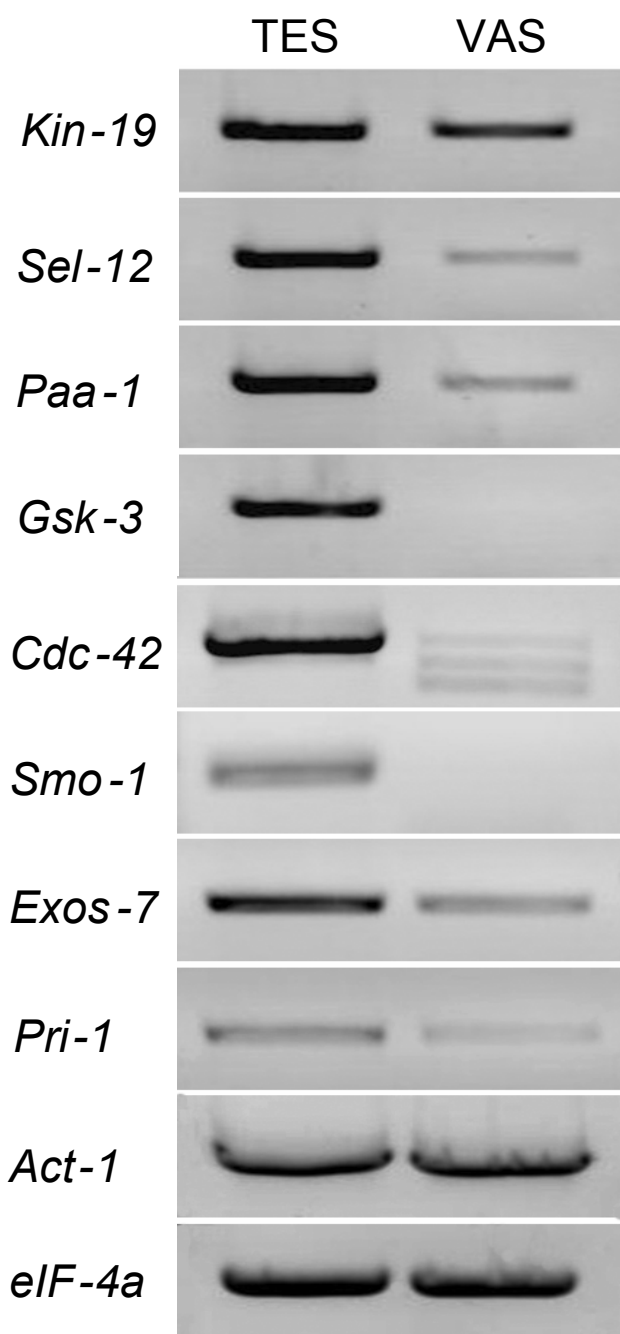

Supplement: Additional file 6 — RT-PCR verification of highly expressed genes in TES. Genes Act-1 and eIF-4A are used as controls. Gene codes are from C. elegans. [file 1471-2164-12-481-S6.PDF]
