# Supplementary material for: Comparative transcriptome sequencing of germline and somatic tissues of the Ascaris suum gonad
Source: BMC Genomics. 2011 Oct 1;12:481. doi: 10.1186/1471-2164-12-481 (PMC3203103; doi:10.1186/1471-2164-12-481)
Supplement: Additional file 5 — Gene/Protein association network for genes highly expressed in TES. The functions of the highlighted gene clusters are as follows: gray, proteasome pathway; yellow, ubiquitin pathway; green, nucleotide metabolism; pink, ribosome members; blue, MSP family. [file 1471-2164-12-481-S5.PDF]

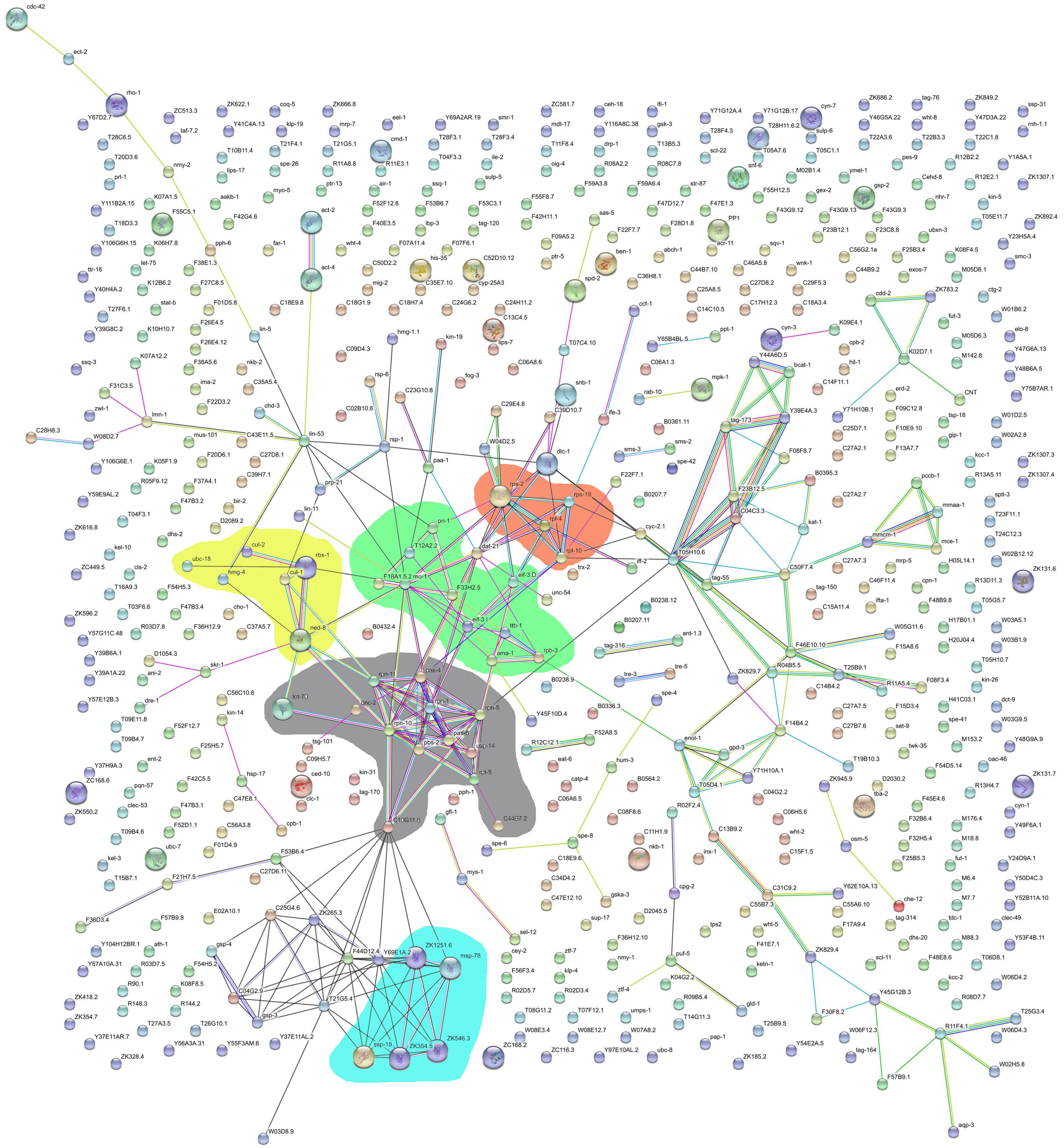

Additional file 5
